# Supplementary figures and images for: Repeated response execution and inhibition alter subjective preferences but do not affect automatic approach and avoidance tendencies toward an object
Source: PeerJ. 2023 Oct 10;11:e16275. doi: 10.7717/peerj.16275 (PMC10573286; doi:10.7717/peerj.16275)

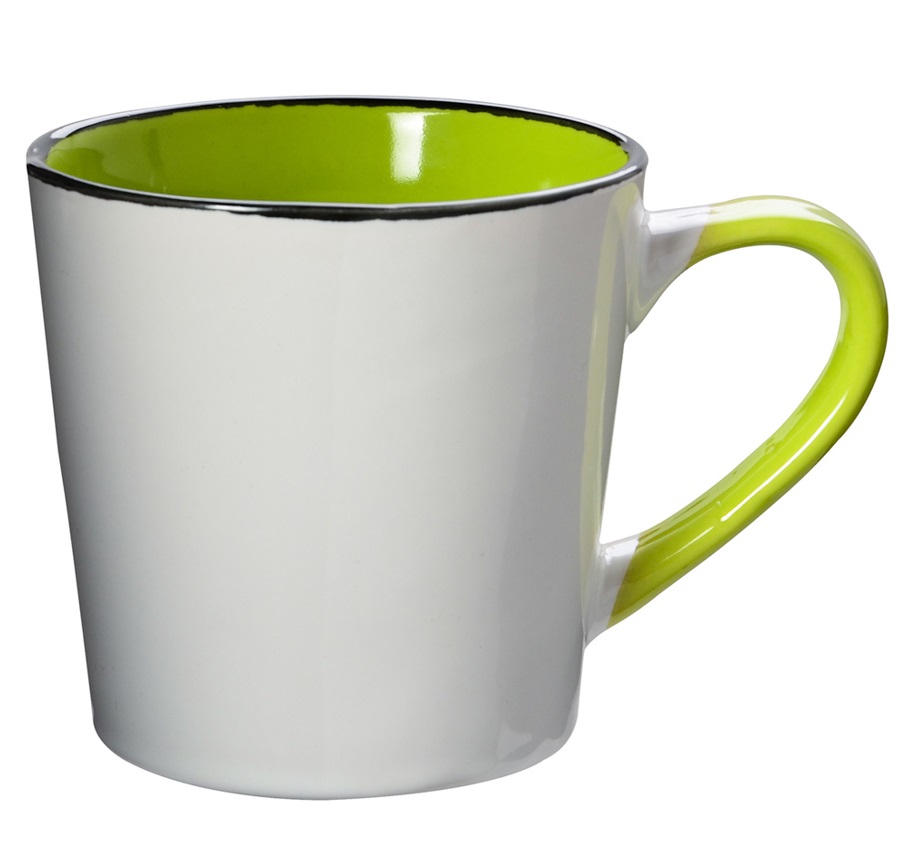

Supplement: Supplemental Information 2 [file peerj-11-16275-s002.jpg]

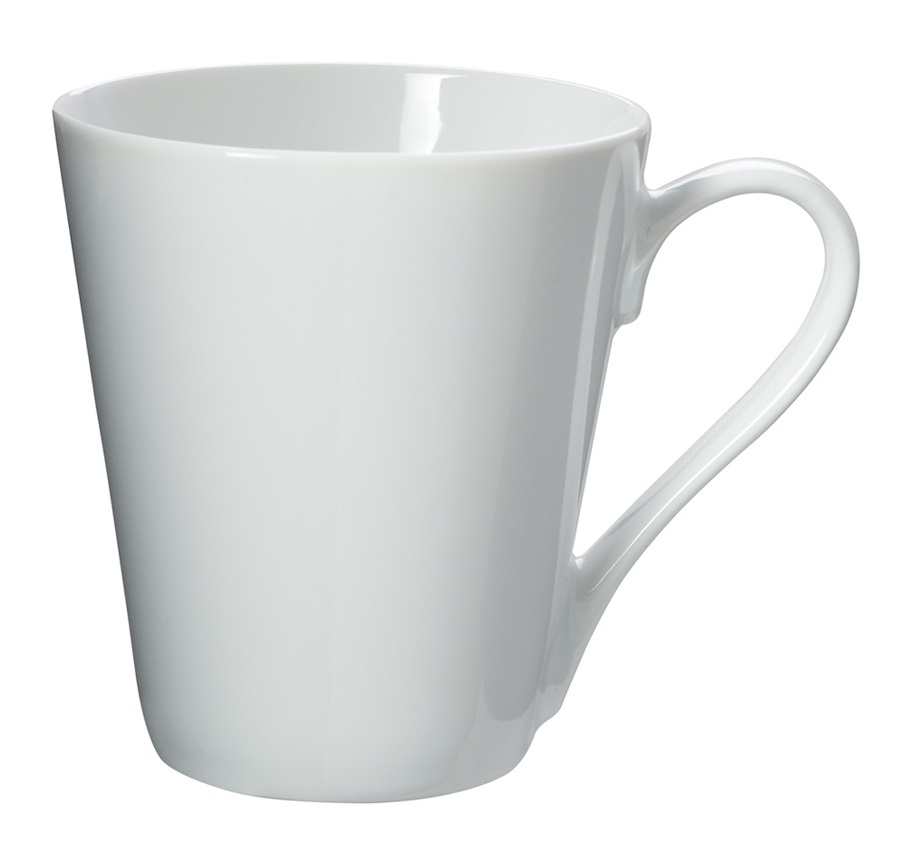

Supplement: Supplemental Information 3 [file peerj-11-16275-s003.jpg]

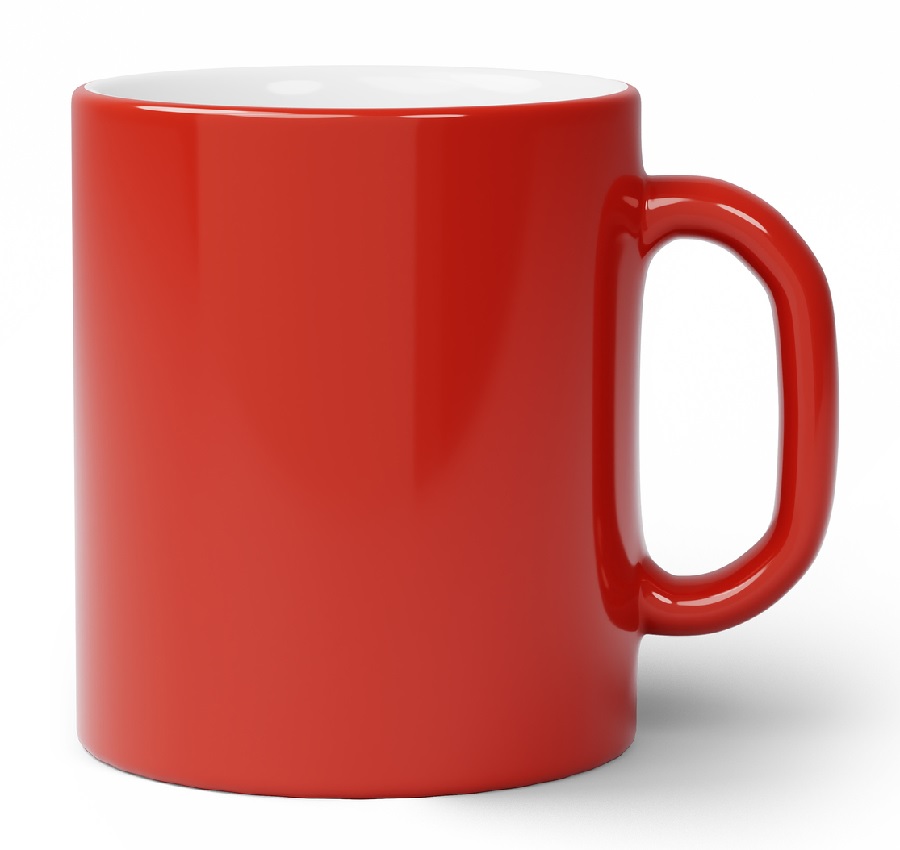

Supplement: Supplemental Information 4 [file peerj-11-16275-s004.jpg]

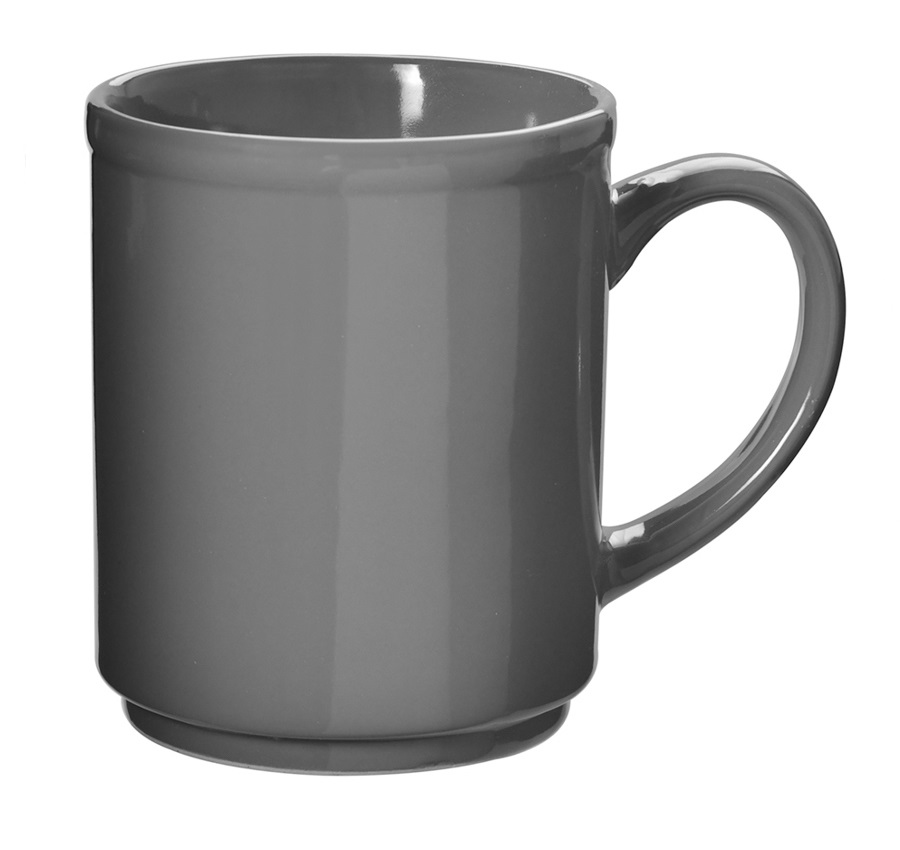

Supplement: Supplemental Information 5 [file peerj-11-16275-s005.jpg]

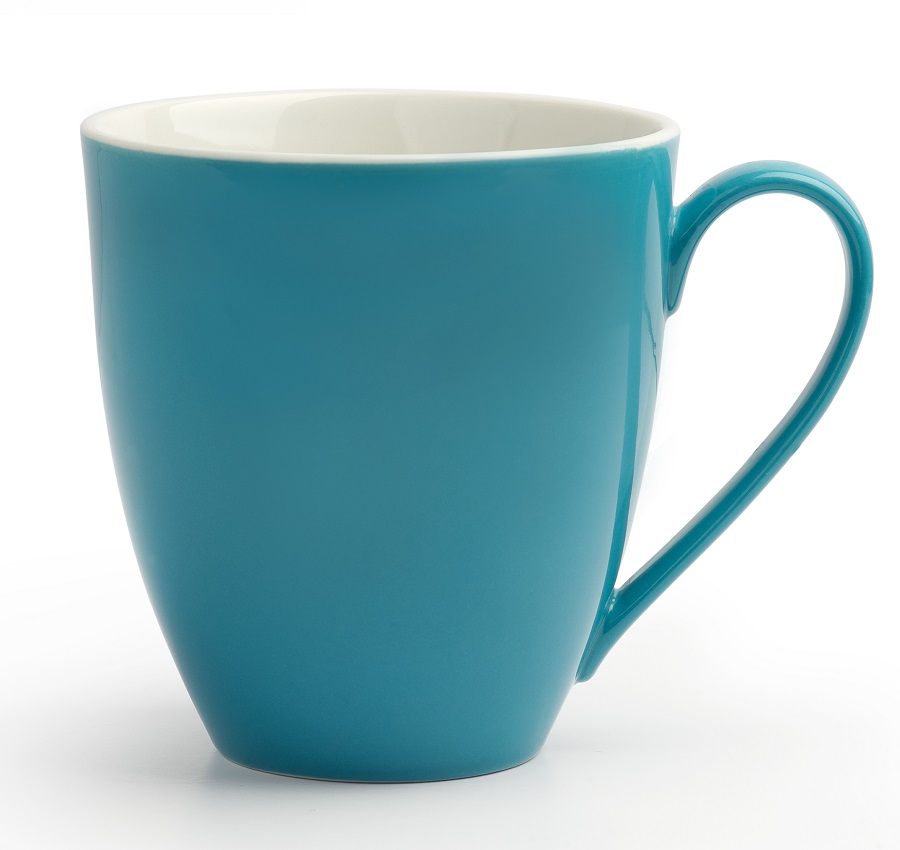

Supplement: Supplemental Information 6 [file peerj-11-16275-s006.jpg]

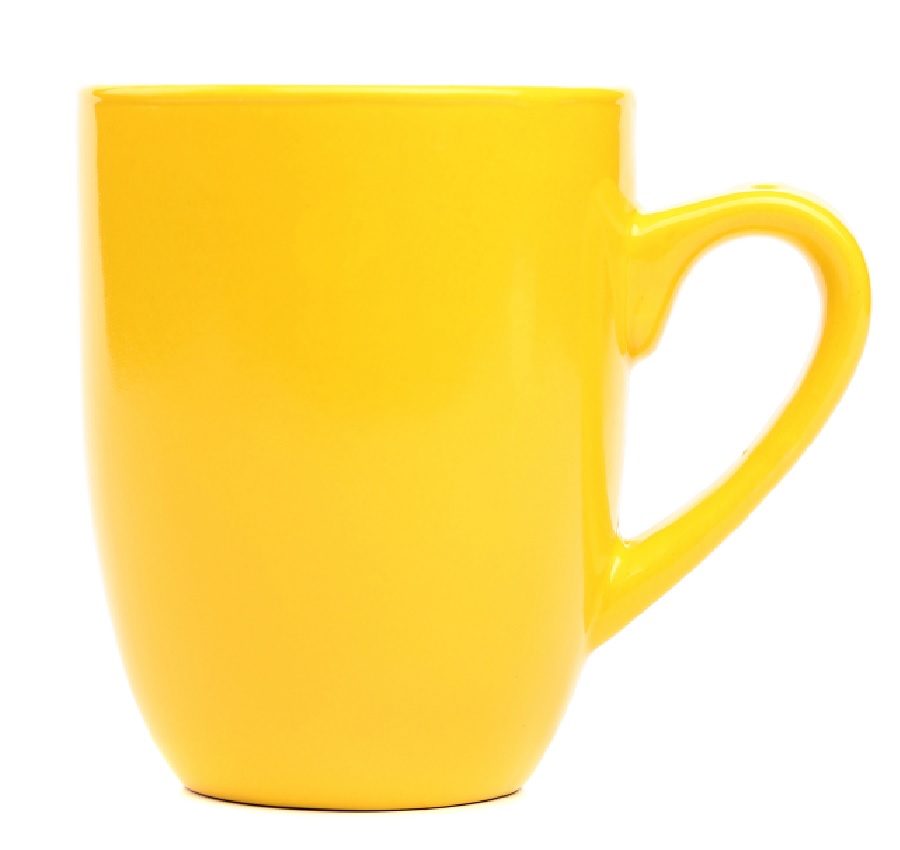

Supplement: Supplemental Information 7 [file peerj-11-16275-s007.jpg]
